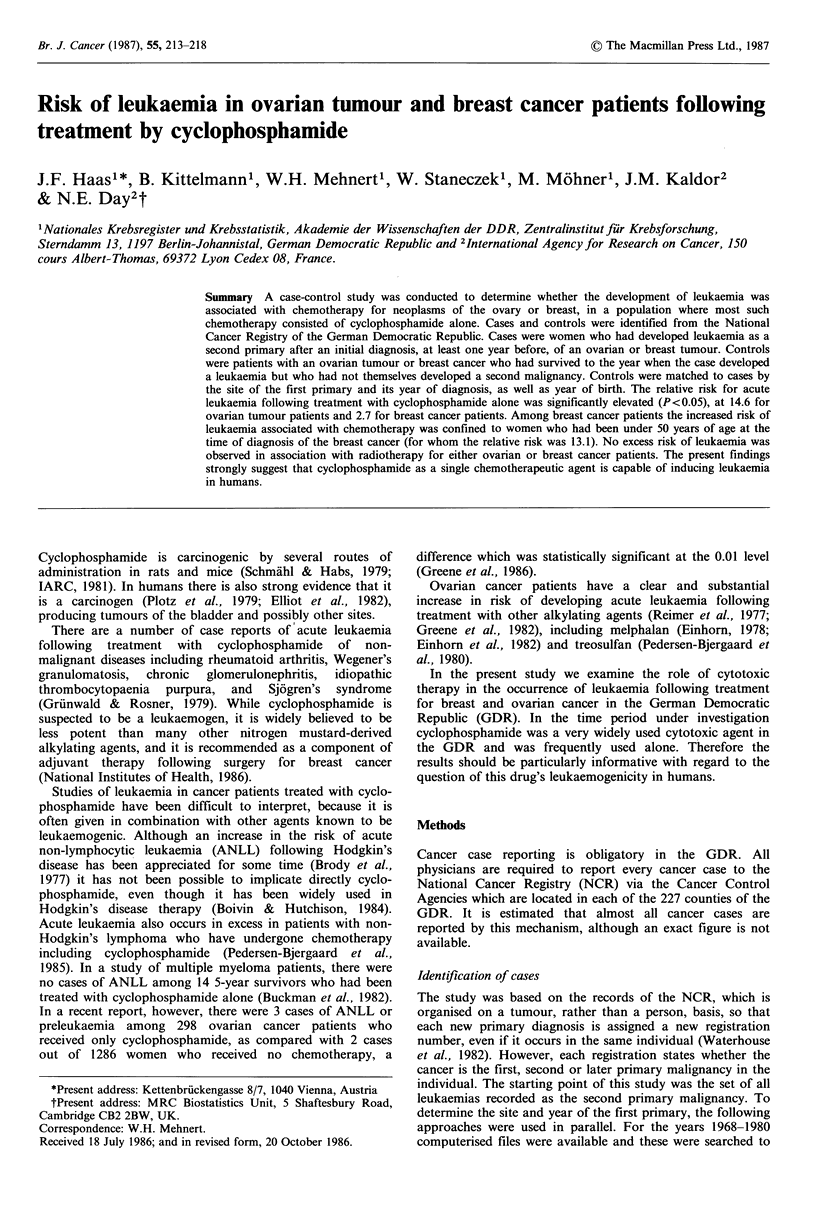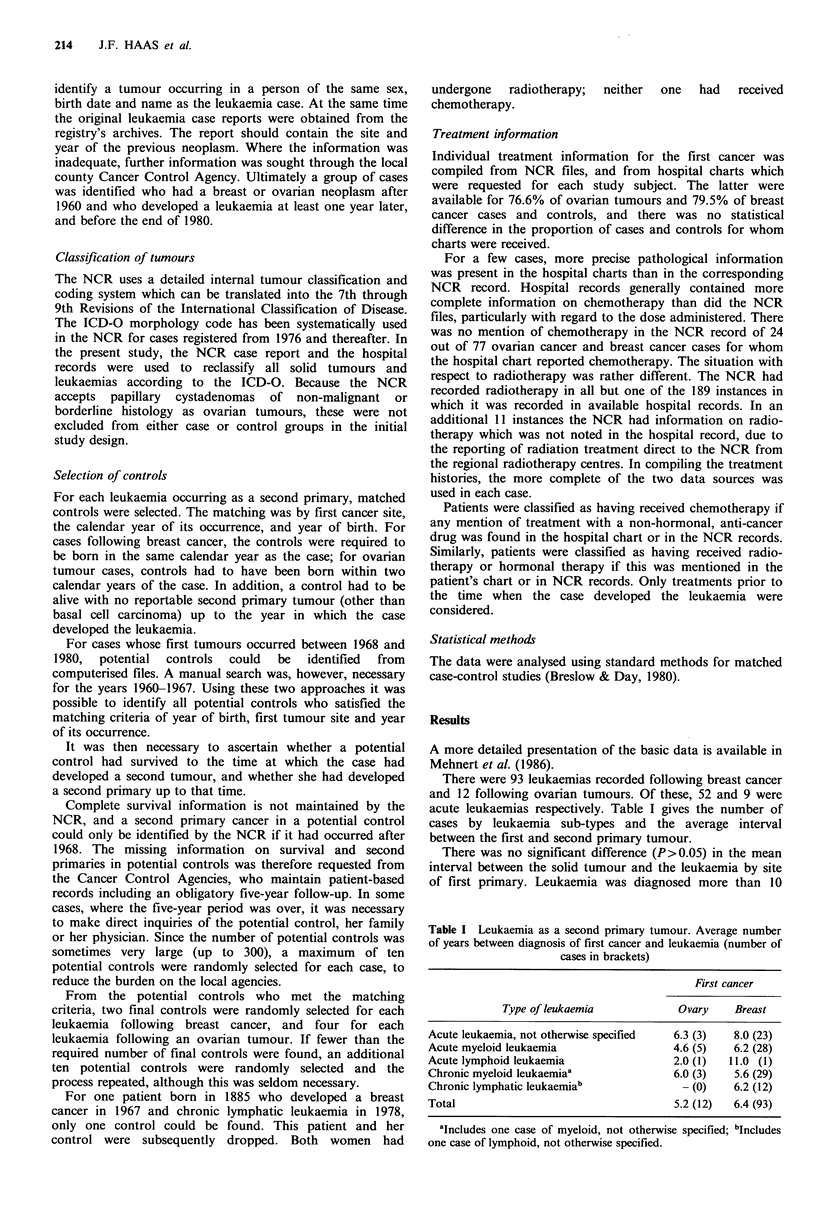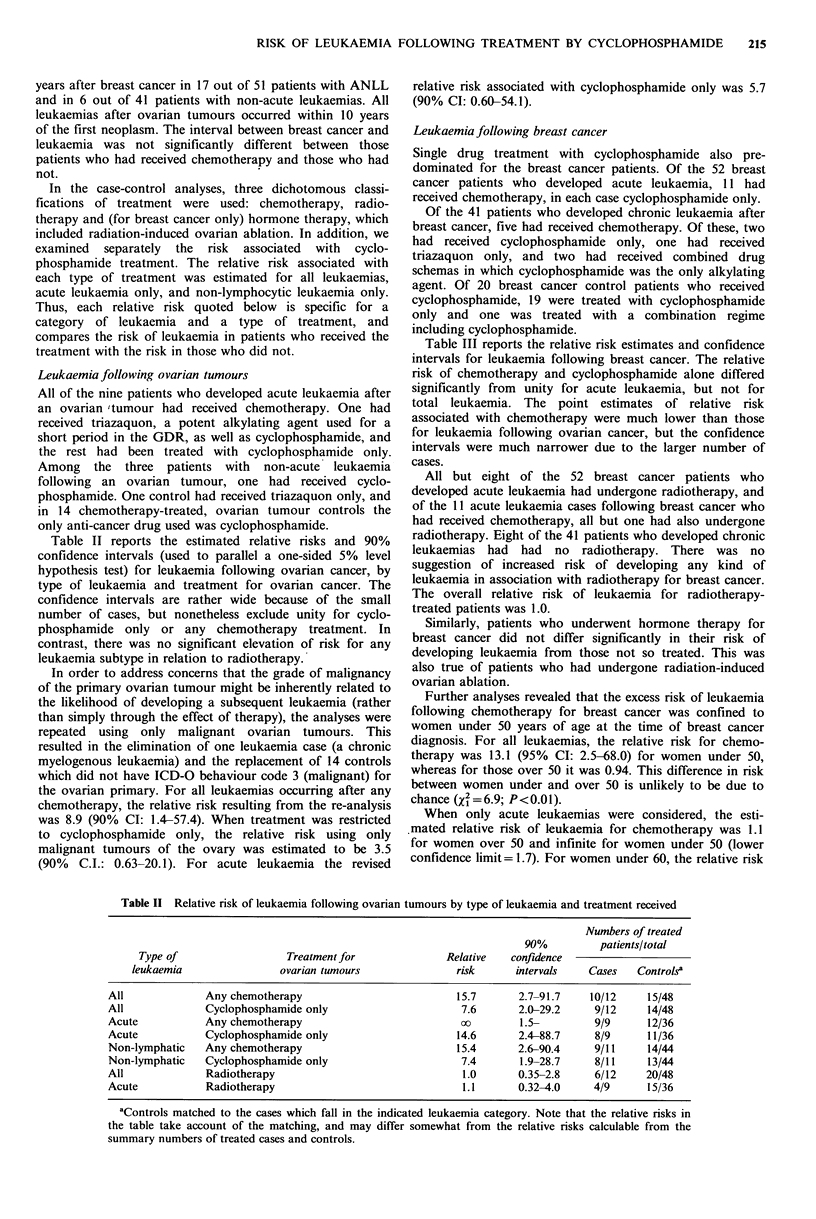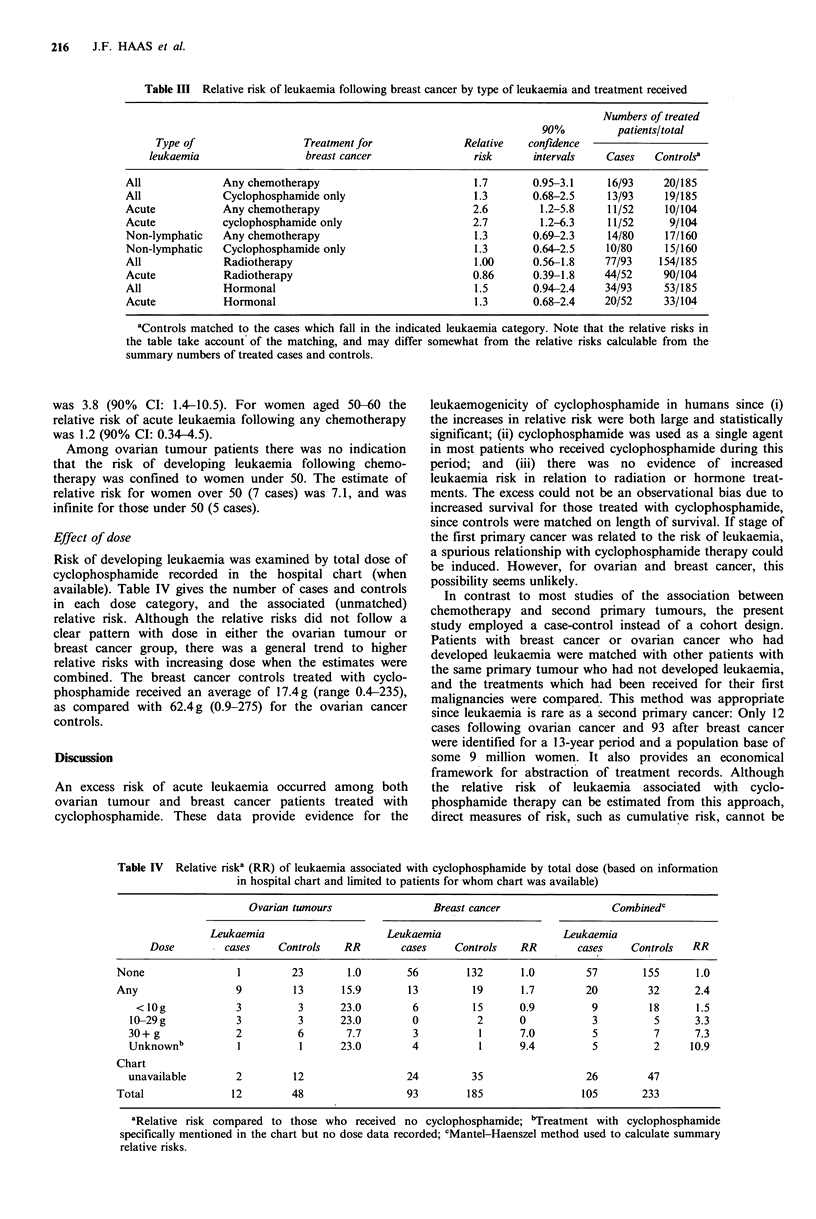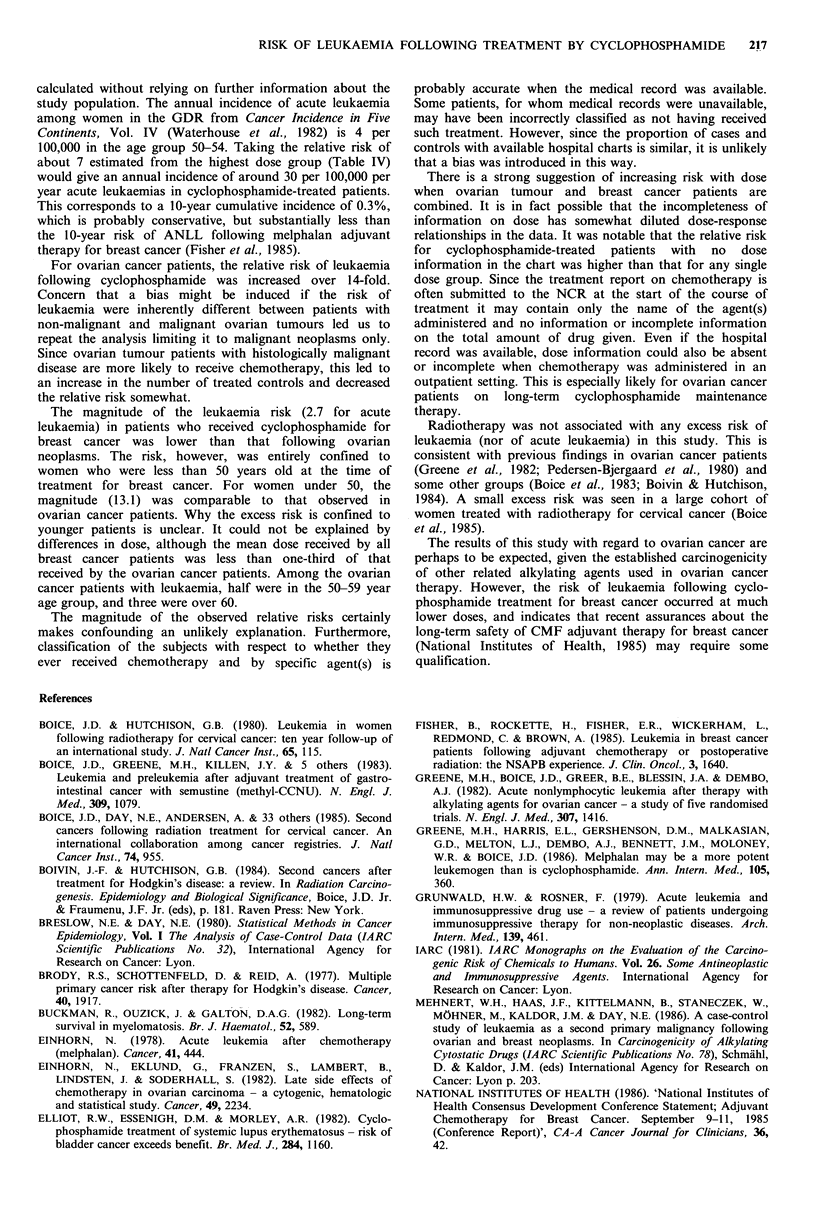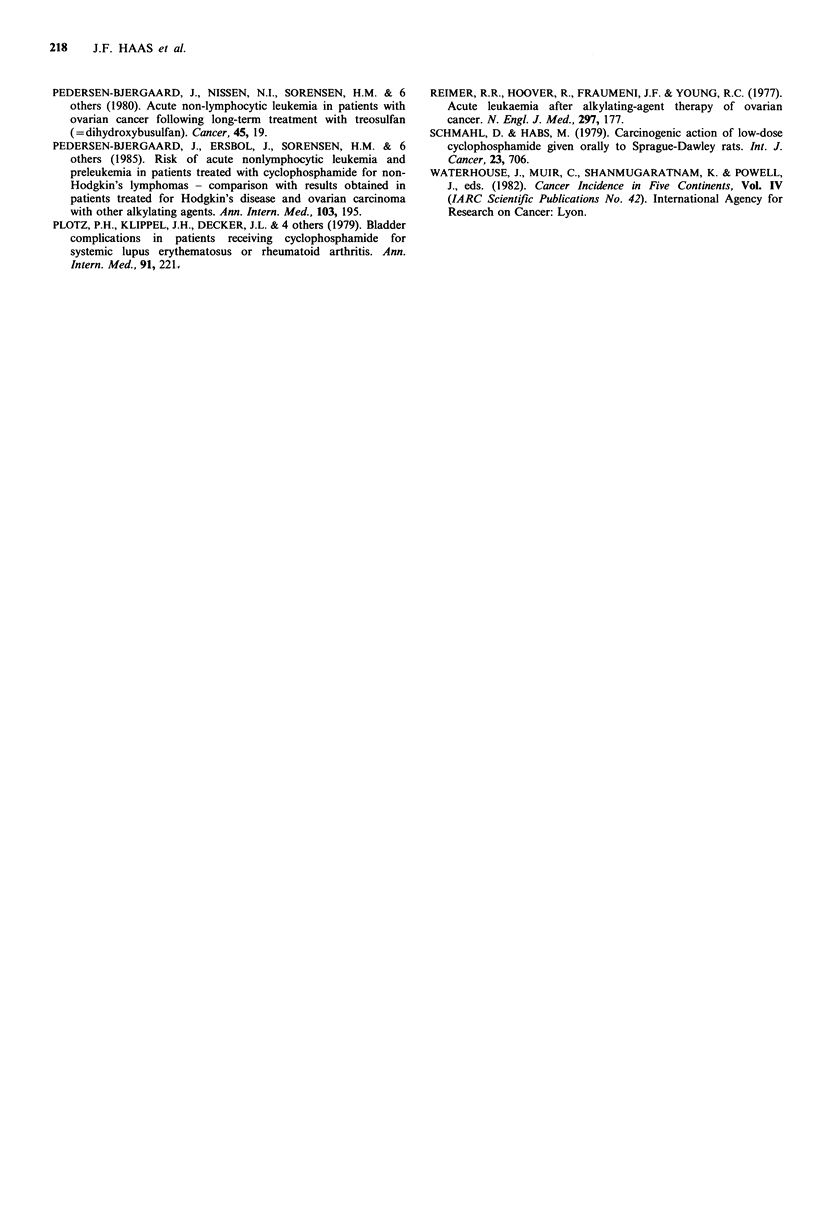# Risk of leukaemia in ovarian tumour and breast cancer patients following treatment by cyclophosphamide.

**DOI:** 10.1038/bjc.1987.40

**Published:** 1987-02

**Authors:** J. F. Haas, B. Kittelmann, W. H. Mehnert, W. Staneczek, M. Möhner, J. M. Kaldor, N. E. Day

## Abstract

A case-control study was conducted to determine whether the development of leukaemia was associated with chemotherapy for neoplasms of the ovary or breast, in a population where most such chemotherapy consisted of cyclophosphamide alone. Cases and controls were identified from the National Cancer Registry of the German Democratic Republic. Cases were women who had developed leukaemia as a second primary after an initial diagnosis, at least one year before, of an ovarian or breast tumour. Controls were patients with an ovarian tumour or breast cancer who had survived to the year when the case developed a leukaemia but who had not themselves developed a second malignancy. Controls were matched to cases by the site of the first primary and its year of diagnosis, as well as year of birth. The relative risk for acute leukaemia following treatment with cyclophosphamide alone was significantly elevated (P less than 0.05), at 14.6 for ovarian tumour patients and 2.7 for breast cancer patients. Among breast cancer patients the increased risk of leukaemia associated with chemotherapy was confined to women who had been under 50 years of age at the time of diagnosis of the breast cancer (for whom the relative risk was 13.1). No excess risk of leukaemia was observed in association with radiotherapy for either ovarian or breast cancer patients. The present findings strongly suggest that cyclophosphamide as a single chemotherapeutic agent is capable of inducing leukaemia in humans.


*Br. J. Cancer* (1987), **55**, 213–218 © The Macmillan Press Ltd., 1987

### Risk of leukaemia in ovarian tumour and breast cancer patients following treatment by cyclophosphamide

J.F. Haas[1]*, B. Kittelmann[1], W.H. Mehnert[1], W. Staneczek[1], M. Möhner[1], J.M. Kaldor[2] & N.E. Day[2]†

[1]*Nationales Krebsregister und Krebsstatistik, Akademie der Wissenschaften der DDR, Zentralinstitut für Krebsforschung, Sterndamm 13, 1197 Berlin-Johannistal, German Democratic Republic and* [2]*International Agency for Research on Cancer, 150 cours Albert-Thomas, 69372 Lyon Cedex 08, France.*

**Summary** A case-control study was conducted to determine whether the development of leukaemia was associated with chemotherapy for neoplasms of the ovary or breast, in a population where most such chemotherapy consisted of cyclophosphamide alone. Cases and controls were identified from the National Cancer Registry of the German Democratic Republic. Cases were women who had developed leukaemia as a second primary after an initial diagnosis, at least one year before, of an ovarian or breast tumour. Controls were patients with an ovarian tumour or breast cancer who had survived to the year when the case developed a leukaemia but who had not themselves developed a second malignancy. Controls were matched to cases by the site of the first primary and its year of diagnosis, as well as year of birth. The relative risk for acute leukaemia following treatment with cyclophosphamide alone was significantly elevated ($P<0.05$), at 14.6 for ovarian tumour patients and 2.7 for breast cancer patients. Among breast cancer patients the increased risk of leukaemia associated with chemotherapy was confined to women who had been under 50 years of age at the time of diagnosis of the breast cancer (for whom the relative risk was 13.1). No excess risk of leukaemia was observed in association with radiotherapy for either ovarian or breast cancer patients. The present findings strongly suggest that cyclophosphamide as a single chemotherapeutic agent is capable of inducing leukaemia in humans.

Cyclophosphamide is carcinogenic by several routes of administration in rats and mice (Schmähl & Habs, 1979; IARC, 1981). In humans there is also strong evidence that it is a carcinogen (Plotz *et al.*, 1979; Elliot *et al.*, 1982), producing tumours of the bladder and possibly other sites.

There are a number of case reports of acute leukaemia following treatment with cyclophosphamide of non-malignant diseases including rheumatoid arthritis, Wegener's granulomatosis, chronic glomerulonephritis, idiopathic thrombocytopaenia purpura, and Sjögren's syndrome (Grünwald & Rosner, 1979). While cyclophosphamide is suspected to be a leukaemogen, it is widely believed to be less potent than many other nitrogen mustard-derived alkylating agents, and it is recommended as a component of adjuvant therapy following surgery for breast cancer (National Institutes of Health, 1986).

Studies of leukaemia in cancer patients treated with cyclophosphamide have been difficult to interpret, because it is often given in combination with other agents known to be leukaemogenic. Although an increase in the risk of acute non-lymphocytic leukaemia (ANLL) following Hodgkin's disease has been appreciated for some time (Brody *et al.*, 1977) it has not been possible to implicate directly cyclophosphamide, even though it has been widely used in Hodgkin's disease therapy (Boivin & Hutchison, 1984). Acute leukaemia also occurs in excess in patients with non-Hodgkin's lymphoma who have undergone chemotherapy including cyclophosphamide (Pedersen-Bjergaard *et al.*, 1985). In a study of multiple myeloma patients, there were no cases of ANLL among 14 5-year survivors who had been treated with cyclophosphamide alone (Buckman *et al.*, 1982). In a recent report, however, there were 3 cases of ANLL or preleukaemia among 298 ovarian cancer patients who received only cyclophosphamide, as compared with 2 cases out of 1286 women who received no chemotherapy, a difference which was statistically significant at the 0.01 level (Greene *et al.*, 1986).

Ovarian cancer patients have a clear and substantial increase in risk of developing acute leukaemia following treatment with other alkylating agents (Reimer *et al.*, 1977; Greene *et al.*, 1982), including melphalan (Einhorn, 1978; Einhorn *et al.*, 1982) and treosulfan (Pedersen-Bjergaard *et al.*, 1980).

In the present study we examine the role of cytotoxic therapy in the occurrence of leukaemia following treatment for breast and ovarian cancer in the German Democratic Republic (GDR). In the time period under investigation cyclophosphamide was a very widely used cytotoxic agent in the GDR and was frequently used alone. Therefore the results should be particularly informative with regard to the question of this drug's leukaemogenicity in humans.

### Methods

Cancer case reporting is obligatory in the GDR. All physicians are required to report every cancer case to the National Cancer Registry (NCR) via the Cancer Control Agencies which are located in each of the 227 counties of the GDR. It is estimated that almost all cancer cases are reported by this mechanism, although an exact figure is not available.

### *Identification of cases*

The study was based on the records of the NCR, which is organised on a tumour, rather than a person, basis, so that each new primary diagnosis is assigned a new registration number, even if it occurs in the same individual (Waterhouse *et al.*, 1982). However, each registration states whether the cancer is the first, second or later primary malignancy in the individual. The starting point of this study was the set of all leukaemias recorded as the second primary malignancy. To determine the site and year of the first primary, the following approaches were used in parallel. For the years 1968–1980 computerised files were available and these were searched to

*Present address: Kettenbrückengasse 8/7, 1040 Vienna, Austria
†Present address: MRC Biostatistics Unit, 5 Shaftesbury Road, Cambridge CB2 2BW, UK.
Correspondence: W.H. Mehnert.
Received 18 July 1986; and in revised form, 20 October 1986.

identify a tumour occurring in a person of the same sex, birth date and name as the leukaemia case. At the same time the original leukaemia case reports were obtained from the registry's archives. The report should contain the site and year of the previous neoplasm. Where the information was inadequate, further information was sought through the local county Cancer Control Agency. Ultimately a group of cases was identified who had a breast or ovarian neoplasm after 1960 and who developed a leukaemia at least one year later, and before the end of 1980.

*Classification of tumours*

The NCR uses a detailed internal tumour classification and coding system which can be translated into the 7th through 9th Revisions of the International Classification of Disease. The ICD-O morphology code has been systematically used in the NCR for cases registered from 1976 and thereafter. In the present study, the NCR case report and the hospital records were used to reclassify all solid tumours and leukaemias according to the ICD-O. Because the NCR accepts papillary cystadenomas of non-malignant or borderline histology as ovarian tumours, these were not excluded from either case or control groups in the initial study design.

*Selection of controls*

For each leukaemia occurring as a second primary, matched controls were selected. The matching was by first cancer site, the calendar year of its occurrence, and year of birth. For cases following breast cancer, the controls were required to be born in the same calendar year as the case; for ovarian tumour cases, controls had to have been born within two calendar years of the case. In addition, a control had to be alive with no reportable second primary tumour (other than basal cell carcinoma) up to the year in which the case developed the leukaemia.

For cases whose first tumours occurred between 1968 and 1980, potential controls could be identified from computerised files. A manual search was, however, necessary for the years 1960–1967. Using these two approaches it was possible to identify all potential controls who satisfied the matching criteria of year of birth, first tumour site and year of its occurrence.

It was then necessary to ascertain whether a potential control had survived to the time at which the case had developed a second tumour, and whether she had developed a second primary up to that time.

Complete survival information is not maintained by the NCR, and a second primary cancer in a potential control could only be identified by the NCR if it had occurred after 1968. The missing information on survival and second primaries in potential controls was therefore requested from the Cancer Control Agencies, who maintain patient-based records including an obligatory five-year follow-up. In some cases, where the five-year period was over, it was necessary to make direct inquiries of the potential control, her family or her physician. Since the number of potential controls was sometimes very large (up to 300), a maximum of ten potential controls were randomly selected for each case, to reduce the burden on the local agencies.

From the potential controls who met the matching criteria, two final controls were randomly selected for each leukaemia following breast cancer, and four for each leukaemia following an ovarian tumour. If fewer than the required number of final controls were found, an additional ten potential controls were randomly selected and the process repeated, although this was seldom necessary.

For one patient born in 1885 who developed a breast cancer in 1967 and chronic lymphatic leukaemia in 1978, only one control could be found. This patient and her control were subsequently dropped. Both women had undergone radiotherapy; neither one had received chemotherapy.

*Treatment information*

Individual treatment information for the first cancer was compiled from NCR files, and from hospital charts which were requested for each study subject. The latter were available for 76.6% of ovarian tumours and 79.5% of breast cancer cases and controls, and there was no statistical difference in the proportion of cases and controls for whom charts were received.

For a few cases, more precise pathological information was present in the hospital charts than in the corresponding NCR record. Hospital records generally contained more complete information on chemotherapy than did the NCR files, particularly with regard to the dose administered. There was no mention of chemotherapy in the NCR record of 24 out of 77 ovarian cancer and breast cancer cases for whom the hospital chart reported chemotherapy. The situation with respect to radiotherapy was rather different. The NCR had recorded radiotherapy in all but one of the 189 instances in which it was recorded in available hospital records. In an additional 11 instances the NCR had information on radiotherapy which was not noted in the hospital record, due to the reporting of radiation treatment direct to the NCR from the regional radiotherapy centres. In compiling the treatment histories, the more complete of the two data sources was used in each case.

Patients were classified as having received chemotherapy if any mention of treatment with a non-hormonal, anti-cancer drug was found in the hospital chart or in the NCR records. Similarly, patients were classified as having received radiotherapy or hormonal therapy if this was mentioned in the patient's chart or in NCR records. Only treatments prior to the time when the case developed the leukaemia were considered.

*Statistical methods*

The data were analysed using standard methods for matched case-control studies (Breslow & Day, 1980).

### Results

A more detailed presentation of the basic data is available in Mehnert *et al.* (1986).

There were 93 leukaemias recorded following breast cancer and 12 following ovarian tumours. Of these, 52 and 9 were acute leukaemias respectively. Table I gives the number of cases by leukaemia sub-types and the average interval between the first and second primary tumour.

There was no significant difference ($P>0.05$) in the mean interval between the solid tumour and the leukaemia by site of first primary. Leukaemia was diagnosed more than 10

**Table I** Leukaemia as a second primary tumour. Average number of years between diagnosis of first cancer and leukaemia (number of cases in brackets)

| | *First cancer* | |
|---|---|---|
| *Type of leukaemia* | *Ovary* | *Breast* |
| Acute leukaemia, not otherwise specified | 6.3 (3) | 8.0 (23) |
| Acute myeloid leukaemia | 4.6 (5) | 6.2 (28) |
| Acute lymphoid leukaemia | 2.0 (1) | 11.0 (1) |
| Chronic myeloid leukaemia[a] | 6.0 (3) | 5.6 (29) |
| Chronic lymphatic leukaemia[b] | – (0) | 6.2 (12) |
| Total | 5.2 (12) | 6.4 (93) |

[a]Includes one case of myeloid, not otherwise specified; [b]Includes one case of lymphoid, not otherwise specified.

years after breast cancer in 17 out of 51 patients with ANLL and in 6 out of 41 patients with non-acute leukaemias. All leukaemias after ovarian tumours occurred within 10 years of the first neoplasm. The interval between breast cancer and leukaemia was not significantly different between those patients who had received chemotherapy and those who had not.

In the case-control analyses, three dichotomous classifications of treatment were used: chemotherapy, radiotherapy and (for breast cancer only) hormone therapy, which included radiation-induced ovarian ablation. In addition, we examined separately the risk associated with cyclophosphamide treatment. The relative risk associated with each type of treatment was estimated for all leukaemias, acute leukaemia only, and non-lymphocytic leukaemia only. Thus, each relative risk quoted below is specific for a category of leukaemia and a type of treatment, and compares the risk of leukaemia in patients who received the treatment with the risk in those who did not.

*Leukaemia following ovarian tumours*

All of the nine patients who developed acute leukaemia after an ovarian tumour had received chemotherapy. One had received triazaquon, a potent alkylating agent used for a short period in the GDR, as well as cyclophosphamide, and the rest had been treated with cyclophosphamide only. Among the three patients with non-acute leukaemia following an ovarian tumour, one had received cyclophosphamide. One control had received triazaquon only, and in 14 chemotherapy-treated, ovarian tumour controls the only anti-cancer drug used was cyclophosphamide.

Table II reports the estimated relative risks and 90% confidence intervals (used to parallel a one-sided 5% level hypothesis test) for leukaemia following ovarian cancer, by type of leukaemia and treatment for ovarian cancer. The confidence intervals are rather wide because of the small number of cases, but nonetheless exclude unity for cyclophosphamide only or any chemotherapy treatment. In contrast, there was no significant elevation of risk for any leukaemia subtype in relation to radiotherapy.

In order to address concerns that the grade of malignancy of the primary ovarian tumour might be inherently related to the likelihood of developing a subsequent leukaemia (rather than simply through the effect of therapy), the analyses were repeated using only malignant ovarian tumours. This resulted in the elimination of one leukaemia case (a chronic myelogenous leukaemia) and the replacement of 14 controls which did not have ICD-O behaviour code 3 (malignant) for the ovarian primary. For all leukaemias occurring after any chemotherapy, the relative risk resulting from the re-analysis was 8.9 (90% CI: 1.4–57.4). When treatment was restricted to cyclophosphamide only, the relative risk using only malignant tumours of the ovary was estimated to be 3.5 (90% C.I.: 0.63–20.1). For acute leukaemia the revised relative risk associated with cyclophosphamide only was 5.7 (90% CI: 0.60–54.1).

*Leukaemia following breast cancer*

Single drug treatment with cyclophosphamide also predominated for the breast cancer patients. Of the 52 breast cancer patients who developed acute leukaemia, 11 had received chemotherapy, in each case cyclophosphamide only.

Of the 41 patients who developed chronic leukaemia after breast cancer, five had received chemotherapy. Of these, two had received cyclophosphamide only, one had received triazaquon only, and two had received combined drug schemas in which cyclophosphamide was the only alkylating agent. Of 20 breast cancer control patients who received cyclophosphamide, 19 were treated with cyclophosphamide only and one was treated with a combination regime including cyclophosphamide.

Table III reports the relative risk estimates and confidence intervals for leukaemia following breast cancer. The relative risk of chemotherapy and cyclophosphamide alone differed significantly from unity for acute leukaemia, but not for total leukaemia. The point estimates of relative risk associated with chemotherapy were much lower than those for leukaemia following ovarian cancer, but the confidence intervals were much narrower due to the larger number of cases.

All but eight of the 52 breast cancer patients who developed acute leukaemia had undergone radiotherapy, and of the 11 acute leukaemia cases following breast cancer who had received chemotherapy, all but one had also undergone radiotherapy. Eight of the 41 patients who developed chronic leukaemias had had no radiotherapy. There was no suggestion of increased risk of developing any kind of leukaemia in association with radiotherapy for breast cancer. The overall relative risk of leukaemia for radiotherapy-treated patients was 1.0.

Similarly, patients who underwent hormone therapy for breast cancer did not differ significantly in their risk of developing leukaemia from those not so treated. This was also true of patients who had undergone radiation-induced ovarian ablation.

Further analyses revealed that the excess risk of leukaemia following chemotherapy for breast cancer was confined to women under 50 years of age at the time of breast cancer diagnosis. For all leukaemias, the relative risk for chemotherapy was 13.1 (95% CI: 2.5–68.0) for women under 50, whereas for those over 50 it was 0.94. This difference in risk between women under and over 50 is unlikely to be due to chance ($\chi^2_1 = 6.9$; $P < 0.01$).

When only acute leukaemias were considered, the estimated relative risk of leukaemia for chemotherapy was 1.1 for women over 50 and infinite for women under 50 (lower confidence limit = 1.7). For women under 60, the relative risk

**Table II** Relative risk of leukaemia following ovarian tumours by type of leukaemia and treatment received

| *Type of leukaemia* | *Treatment for ovarian tumours* | *Relative risk* | *90% confidence intervals* | *Numbers of treated patients/total* | |
|---|---|---|---|---|---|
| | | | | *Cases* | *Controls*[a] |
| All | Any chemotherapy | 15.7 | 2.7–91.7 | 10/12 | 15/48 |
| All | Cyclophosphamide only | 7.6 | 2.0–29.2 | 9/12 | 14/48 |
| Acute | Any chemotherapy | ∞ | 1.5– | 9/9 | 12/36 |
| Acute | Cyclophosphamide only | 14.6 | 2.4–88.7 | 8/9 | 11/36 |
| Non-lymphatic | Any chemotherapy | 15.4 | 2.6–90.4 | 9/11 | 14/44 |
| Non-lymphatic | Cyclophosphamide only | 7.4 | 1.9–28.7 | 8/11 | 13/44 |
| All | Radiotherapy | 1.0 | 0.35–2.8 | 6/12 | 20/48 |
| Acute | Radiotherapy | 1.1 | 0.32–4.0 | 4/9 | 15/36 |

[a]Controls matched to the cases which fall in the indicated leukaemia category. Note that the relative risks in the table take account of the matching, and may differ somewhat from the relative risks calculable from the summary numbers of treated cases and controls.

**Table III** Relative risk of leukaemia following breast cancer by type of leukaemia and treatment received

| *Type of leukaemia* | *Treatment for breast cancer* | *Relative risk* | *90% confidence intervals* | *Numbers of treated patients/total* | |
|---|---|---|---|---|---|
| | | | | *Cases* | *Controls*[a] |
| All | Any chemotherapy | 1.7 | 0.95–3.1 | 16/93 | 20/185 |
| All | Cyclophosphamide only | 1.3 | 0.68–2.5 | 13/93 | 19/185 |
| Acute | Any chemotherapy | 2.6 | 1.2–5.8 | 11/52 | 10/104 |
| Acute | cyclophosphamide only | 2.7 | 1.2–6.3 | 11/52 | 9/104 |
| Non-lymphatic | Any chemotherapy | 1.3 | 0.69–2.3 | 14/80 | 17/160 |
| Non-lymphatic | Cyclophosphamide only | 1.3 | 0.64–2.5 | 10/80 | 15/160 |
| All | Radiotherapy | 1.00 | 0.56–1.8 | 77/93 | 154/185 |
| Acute | Radiotherapy | 0.86 | 0.39–1.8 | 44/52 | 90/104 |
| All | Hormonal | 1.5 | 0.94–2.4 | 34/93 | 53/185 |
| Acute | Hormonal | 1.3 | 0.68–2.4 | 20/52 | 33/104 |

[a]Controls matched to the cases which fall in the indicated leukaemia category. Note that the relative risks in the table take account of the matching, and may differ somewhat from the relative risks calculable from the summary numbers of treated cases and controls.

was 3.8 (90% CI: 1.4–10.5). For women aged 50–60 the relative risk of acute leukaemia following any chemotherapy was 1.2 (90% CI: 0.34–4.5).

Among ovarian tumour patients there was no indication that the risk of developing leukaemia following chemotherapy was confined to women under 50. The estimate of relative risk for women over 50 (7 cases) was 7.1, and was infinite for those under 50 (5 cases).

### *Effect of dose*

Risk of developing leukaemia was examined by total dose of cyclophosphamide recorded in the hospital chart (when available). Table IV gives the number of cases and controls in each dose category, and the associated (unmatched) relative risk. Although the relative risks did not follow a clear pattern with dose in either the ovarian tumour or breast cancer group, there was a general trend to higher relative risks with increasing dose when the estimates were combined. The breast cancer controls treated with cyclophosphamide received an average of 17.4 g (range 0.4–235), as compared with 62.4 g (0.9–275) for the ovarian cancer controls.

### Discussion

An excess risk of acute leukaemia occurred among both ovarian tumour and breast cancer patients treated with cyclophosphamide. These data provide evidence for the leukaemogenicity of cyclophosphamide in humans since (i) the increases in relative risk were both large and statistically significant; (ii) cyclophosphamide was used as a single agent in most patients who received cyclophosphamide during this period; and (iii) there was no evidence of increased leukaemia risk in relation to radiation or hormone treatments. The excess could not be an observational bias due to increased survival for those treated with cyclophosphamide, since controls were matched on length of survival. If stage of the first primary cancer was related to the risk of leukaemia, a spurious relationship with cyclophosphamide therapy could be induced. However, for ovarian and breast cancer, this possibility seems unlikely.

In contrast to most studies of the association between chemotherapy and second primary tumours, the present study employed a case-control instead of a cohort design. Patients with breast cancer or ovarian cancer who had developed leukaemia were matched with other patients with the same primary tumour who had not developed leukaemia, and the treatments which had been received for their first malignancies were compared. This method was appropriate since leukaemia is rare as a second primary cancer: Only 12 cases following ovarian cancer and 93 after breast cancer were identified for a 13-year period and a population base of some 9 million women. It also provides an economical framework for abstraction of treatment records. Although the relative risk of leukaemia associated with cyclophosphamide therapy can be estimated from this approach, direct measures of risk, such as cumulative risk, cannot be

**Table IV** Relative risk[a] (RR) of leukaemia associated with cyclophosphamide by total dose (based on information in hospital chart and limited to patients for whom chart was available)

| | *Ovarian tumours* | | | *Breast cancer* | | | *Combined*[c] | | |
|---|---|---|---|---|---|---|---|---|---|
| *Dose* | *Leukaemia cases* | *Controls* | *RR* | *Leukaemia cases* | *Controls* | *RR* | *Leukaemia cases* | *Controls* | *RR* |
| None | 1 | 23 | 1.0 | 56 | 132 | 1.0 | 57 | 155 | 1.0 |
| Any | 9 | 13 | 15.9 | 13 | 19 | 1.7 | 20 | 32 | 2.4 |
| <10 g | 3 | 3 | 23.0 | 6 | 15 | 0.9 | 9 | 18 | 1.5 |
| 10–29 g | 3 | 3 | 23.0 | 0 | 2 | 0 | 3 | 5 | 3.3 |
| 30+ g | 2 | 6 | 7.7 | 3 | 1 | 7.0 | 5 | 7 | 7.3 |
| Unknown[b] | 1 | 1 | 23.0 | 4 | 1 | 9.4 | 5 | 2 | 10.9 |
| Chart unavailable | 2 | 12 | | 24 | 35 | | 26 | 47 | |
| Total | 12 | 48 | | 93 | 185 | | 105 | 233 | |

[a]Relative risk compared to those who received no cyclophosphamide; [b]Treatment with cyclophosphamide specifically mentioned in the chart but no dose data recorded; [c]Mantel–Haenszel method used to calculate summary relative risks.

calculated without relying on further information about the study population. The annual incidence of acute leukaemia among women in the GDR from *Cancer Incidence in Five Continents*, Vol. IV (Waterhouse *et al.*, 1982) is 4 per 100,000 in the age group 50–54. Taking the relative risk of about 7 estimated from the highest dose group (Table IV) would give an annual incidence of around 30 per 100,000 per year acute leukaemias in cyclophosphamide-treated patients. This corresponds to a 10-year cumulative incidence of 0.3%, which is probably conservative, but substantially less than the 10-year risk of ANLL following melphalan adjuvant therapy for breast cancer (Fisher *et al.*, 1985).

For ovarian cancer patients, the relative risk of leukaemia following cyclophosphamide was increased over 14-fold. Concern that a bias might be induced if the risk of leukaemia were inherently different between patients with non-malignant and malignant ovarian tumours led us to repeat the analysis limiting it to malignant neoplasms only. Since ovarian tumour patients with histologically malignant disease are more likely to receive chemotherapy, this led to an increase in the number of treated controls and decreased the relative risk somewhat.

The magnitude of the leukaemia risk (2.7 for acute leukaemia) in patients who received cyclophosphamide for breast cancer was lower than that following ovarian neoplasms. The risk, however, was entirely confined to women who were less than 50 years old at the time of treatment for breast cancer. For women under 50, the magnitude (13.1) was comparable to that observed in ovarian cancer patients. Why the excess risk is confined to younger patients is unclear. It could not be explained by differences in dose, although the mean dose received by all breast cancer patients was less than one-third of that received by the ovarian cancer patients. Among the ovarian cancer patients with leukaemia, half were in the 50–59 year age group, and three were over 60.

The magnitude of the observed relative risks certainly makes confounding an unlikely explanation. Furthermore, classification of the subjects with respect to whether they ever received chemotherapy and by specific agent(s) is probably accurate when the medical record was available. Some patients, for whom medical records were unavailable, may have been incorrectly classified as not having received such treatment. However, since the proportion of cases and controls with available hospital charts is similar, it is unlikely that a bias was introduced in this way.

There is a strong suggestion of increasing risk with dose when ovarian tumour and breast cancer patients are combined. It is in fact possible that the incompleteness of information on dose has somewhat diluted dose-response relationships in the data. It was notable that the relative risk for cyclophosphamide-treated patients with no dose information in the chart was higher than that for any single dose group. Since the treatment report on chemotherapy is often submitted to the NCR at the start of the course of treatment it may contain only the name of the agent(s) administered and no information or incomplete information on the total amount of drug given. Even if the hospital record was available, dose information could also be absent or incomplete when chemotherapy was administered in an outpatient setting. This is especially likely for ovarian cancer patients on long-term cyclophosphamide maintenance therapy.

Radiotherapy was not associated with any excess risk of leukaemia (nor of acute leukaemia) in this study. This is consistent with previous findings in ovarian cancer patients (Greene *et al.*, 1982; Pedersen-Bjergaard *et al.*, 1980) and some other groups (Boice *et al.*, 1983; Boivin & Hutchison, 1984). A small excess risk was seen in a large cohort of women treated with radiotherapy for cervical cancer (Boice *et al.*, 1985).

The results of this study with regard to ovarian cancer are perhaps to be expected, given the established carcinogenicity of other related alkylating agents used in ovarian cancer therapy. However, the risk of leukaemia following cyclophosphamide treatment for breast cancer occurred at much lower doses, and indicates that recent assurances about the long-term safety of CMF adjuvant therapy for breast cancer (National Institutes of Health, 1985) may require some qualification.

### References

BOICE, J.D. & HUTCHISON, G.B. (1980). Leukemia in women following radiotherapy for cervical cancer: ten year follow-up of an international study. *J. Natl Cancer Inst.*, **65**, 115.

BOICE, J.D., GREENE, M.H., KILLEN, J.Y. & 5 others (1983). Leukemia and preleukemia after adjuvant treatment of gastro-intestinal cancer with semustine (methyl-CCNU). *N. Engl. J. Med.*, **309**, 1079.

BOICE, J.D., DAY, N.E., ANDERSEN, A. & 33 others (1985). Second cancers following radiation treatment for cervical cancer. An international collaboration among cancer registries. *J. Natl Cancer Inst.*, **74**, 955.

BOIVIN, J.-F. & HUTCHISON, G.B. (1984). Second cancers after treatment for Hodgkin's disease: a review. In *Radiation Carcinogenesis. Epidemiology and Biological Significance*, Boice, J.D. Jr. & Fraumenu, J.F. Jr. (eds), p. 181. Raven Press: New York.

BRESLOW, N.E. & DAY, N.E. (1980). *Statistical Methods in Cancer Epidemiology*, **Vol. I** *The Analysis of Case-Control Data* (*IARC Scientific Publications No. 32*), International Agency for Research on Cancer: Lyon.

BRODY, R.S., SCHOTTENFELD, D. & REID, A. (1977). Multiple primary cancer risk after therapy for Hodgkin's disease. *Cancer*, **40**, 1917.

BUCKMAN, R., OUZICK, J. & GALTON, D.A.G. (1982). Long-term survival in myelomatosis. *Br. J. Haematol.*, **52**, 589.

EINHORN, N. (1978). Acute leukemia after chemotherapy (melphalan). *Cancer*, **41**, 444.

EINHORN, N., EKLUND, G., FRANZEN, S., LAMBERT, B., LINDSTEN, J. & SODERHALL, S. (1982). Late side effects of chemotherapy in ovarian carcinoma – a cytogenic, hematologic and statistical study. *Cancer*, **49**, 2234.

ELLIOT, R.W., ESSENIGH, D.M. & MORLEY, A.R. (1982). Cyclophosphamide treatment of systemic lupus erythematosus – risk of bladder cancer exceeds benefit. *Br. Med. J.*, **284**, 1160.

FISHER, B., ROCKETTE, H., FISHER, E.R., WICKERHAM, L., REDMOND, C. & BROWN, A. (1985). Leukemia in breast cancer patients following adjuvant chemotherapy or postoperative radiation: the NSAPB experience. *J. Clin. Oncol.*, **3**, 1640.

GREENE, M.H., BOICE, J.D., GREER, B.E., BLESSIN, J.A. & DEMBO, A.J. (1982). Acute nonlymphocytic leukemia after therapy with alkylating agents for ovarian cancer – a study of five randomised trials. *N. Engl. J. Med.*, **307**, 1416.

GREENE, M.H., HARRIS, E.L., GERSHENSON, D.M., MALKASIAN, G.D., MELTON, L.J., DEMBO, A.J., BENNETT, J.M., MOLONEY, W.R. & BOICE, J.D. (1986). Melphalan may be a more potent leukemogen than is cyclophosphamide. *Ann. Intern. Med.*, **105**, 360.

GRUNWALD, H.W. & ROSNER, F. (1979). Acute leukemia and immunosuppressive drug use – a review of patients undergoing immunosuppressive therapy for non-neoplastic diseases. *Arch. Intern. Med.*, **139**, 461.

IARC (1981). *IARC Monographs on the Evaluation of the Carcinogenic Risk of Chemicals to Humans*. **Vol. 26.** *Some Antineoplastic and Immunosuppressive Agents*. International Agency for Research on Cancer: Lyon.

MEHNERT, W.H., HAAS, J.F., KITTELMANN, B., STANECZEK, W., MÖHNER, M., KALDOR, J.M. & DAY, N.E. (1986). A case-control study of leukaemia as a second primary malignancy following ovarian and breast neoplasms. In *Carcinogenicity of Alkylating Cytostatic Drugs* (*IARC Scientific Publications No. 78*), Schmähl, D. & Kaldor, J.M. (eds) International Agency for Research on Cancer: Lyon p. 203.

NATIONAL INSTITUTES OF HEALTH (1986). 'National Institutes of Health Consensus Development Conference Statement; Adjuvant Chemotherapy for Breast Cancer. September 9–11, 1985 (Conference Report)', *CA-A Cancer Journal for Clinicians*, **36**, 42.

PEDERSEN-BJERGAARD, J., NISSEN, N.I., SORENSEN, H.M. & 6 others (1980). Acute non-lymphocytic leukemia in patients with ovarian cancer following long-term treatment with treosulfan (=dihydroxybusulfan). *Cancer*, **45**, 19.

PEDERSEN-BJERGAARD, J., ERSBOL, J., SORENSEN, H.M. & 6 others (1985). Risk of acute nonlymphocytic leukemia and preleukemia in patients treated with cyclophosphamide for non-Hodgkin's lymphomas – comparison with results obtained in patients treated for Hodgkin's disease and ovarian carcinoma with other alkylating agents. *Ann. Intern. Med.*, **103**, 195.

PLOTZ, P.H., KLIPPEL, J.H., DECKER, J.L. & 4 others (1979). Bladder complications in patients receiving cyclophosphamide for systemic lupus erythematosus or rheumatoid arthritis. *Ann. Intern. Med.*, **91**, 221.

REIMER, R.R., HOOVER, R., FRAUMENI, J.F. & YOUNG, R.C. (1977). Acute leukaemia after alkylating-agent therapy of ovarian cancer. *N. Engl. J. Med.*, **297**, 177.

SCHMAHL, D. & HABS, M. (1979). Carcinogenic action of low-dose cyclophosphamide given orally to Sprague-Dawley rats. *Int. J. Cancer*, **23**, 706.

WATERHOUSE, J., MUIR, C., SHANMUGARATNAM, K. & POWELL, J., eds. (1982). *Cancer Incidence in Five Continents*, **Vol. IV** (*IARC Scientific Publications No. 42*). International Agency for Research on Cancer: Lyon.